# Activation of epiplexus macrophages in hydrocephalus caused by subarachnoid hemorrhage and thrombin

**DOI:** 10.1111/cns.13203

**Published:** 2019-08-21

**Authors:** Yingfeng Wan, Ya Hua, Hugh J. L. Garton, Nemanja Novakovic, Richard F. Keep, Guohua Xi

**Affiliations:** ^1^ Department of Neurosurgery University of Michigan Ann Arbor MI USA; ^2^ Department of Neurosurgery, Sir Run Run Shaw Hospital Zhejiang University Hangzhou China

**Keywords:** epiplexus cells, hydrocephalus, subarachnoid hemorrhage, thrombin

## Abstract

**Aims:**

We have found that hydrocephalus development in spontaneously hypertensive rats was associated with activation of epiplexus cells. The current study examined whether epiplexus cell activation occurs in a rat subarachnoid hemorrhage (SAH), whether activation would be greater in a subset of rats that developed hydrocephalus and the potential role of thrombin in epiplexus cell activation.

**Methods:**

There were two parts in this study. First, an endovascular perforation was performed in rats to induce SAH. Second, rats received an intraventricular infusion of either thrombin or saline. Magnetic resonance imaging was used to measure the ventricular volumes. Immunofluorescence and immunohistochemistry were used to study epiplexus cell activation.

**Results:**

Iba‐1, OX‐6, and CD68 were expressed in the epiplexus cells of the choroid plexus in sham‐operated rats. SAH increased Iba‐1 and CD68 immunoreactivity in epiplexus cells in addition to an increase in Iba‐1‐positive cell soma size. Those effects were greater in rats that developed hydrocephalus. Intraventricular thrombin mimicked the effects of SAH on epiplexus cell activation and hydrocephalus.

**Conclusion:**

This study supports the concept that epiplexus cell activation is associated with hydrocephalus development. Epiplexus cell activation may be in response to thrombin production after hemorrhage, and it may be a therapeutic target.

## 1 INTRODUCTION

There is a well‐established association between an intraventricular hemorrhage (IVH) and hydrocephalus development. However, the precise mechanisms remain unclear. Understanding the mechanisms may lead to changes in how we manage patients with hydrocephalus, especially because we currently experience poor clinical outcomes in patients that develop hydrocephalus after IVH.^1^ Additionally, it is known that hydrocephalus and IVH are both independent predictors of poor outcome in spontaneous ICH.^2^ Patients with concurrent IVH and ICH development had a favorable outcome of 15% compared with 31% with ICH alone. Hydrocephalus, which develops in 55% of patients with IVH, further decreased the chance of a favorable outcome to 11.5%.^3^ Another independent prognostic factor for unfavorable outcomes is IVH in patients with subarachnoid hemorrhage (SAH). 20%‐30% of such patients develop acute hydrocephalus.^4^ The increased intracranial pressure associated with hydrocephalus correlates with poorer prognosis in patients with aneurysmal SAH.^5^, ^6^

Hydrocephalus development after IVH is not only a major complication in adult patients with ICH and SAH, but also in premature infants. More than 12 000 premature infants in the United States develop hydrocephalus after IVH every year. IVH is an independent poor prognostic predictor for neonates after a germinal matrix hemorrhage.^7^

The choroid plexus is a hyper‐vascularized tissue in the brain ventricle system which predominantly functions to secrete CSF. Epiplexus cells, also named “Kolmer cells,” were initially detailed in 1921 by Kolmer. These cells have primarily been examined using immunohistochemistry and electron microscopy.^8^ They exhibit diverse morphologies, including round, polar, and stellate. They are macrophages of monocytic origin in the brain ventricle system considered to have an immunologic role due to their involvement with antigen presentation, nitric oxide (NO) production, phagocytosis of various foreign bodies, and iron accumulation.^9^

It is generally accepted that inflammation has a role in hydrocephalus development.^10^ Recent evidence indicates that inflammatory signaling at the choroid plexus is involved in CSF hypersecretion and hydrocephalus development after cerebral hemorrhage.^11^ We recently found that epiplexus cell activation at the choroid plexus occurs during hydrocephalus development that naturally occurs in spontaneous hypertensive rats.^12^ Therefore, the current study was undertaken to examine whether epiplexus cell activation occurs in a rat model of SAH, whether the degree of activation would be greater in those rats that develop hydrocephalus after SAH, and whether the effects of SAH could be mimicked by intraventricular thrombin. Thrombin is produced during clot formation, it is known to activate microglia/macrophages,^13^ and we have recently shown it can induce hydrocephalus in rats.^14^

## 2 MATERIALS AND METHODS

### 2.1 Animal preparation

All animal protocols were approved by the University of Michigan Committee on the Use and Care of Animals. Standard 12:12 light‐dark conditions were used to house the rats, and they were allowed free access to water and food. The experiments used a total of 70 male adult Sprague Dawley rats (2‐3 month old, Charles River Laboratories).

### 2.2 Experiment groups

The study had two parts. In the first part, rats were randomly assigned into two groups: a sham operation (n = 12) or SAH induction (n = 25). Eight rats died within 24 hours after SAH induction. Surviving rats were sacrificed at 24 hours after the operations. The SAH group was further divided into two groups based on MRI scanning for ventricular volume: SAH with hydrocephalus (SAH with HC, n = 15) and SAH without hydrocephalus (SAH w/o HC, n = 10). In the second part, 25 rats were injected intracerebroventricularly with 50 µL (3U) of rat thrombin (Sigma‐Aldrich) in saline (n = 11) or 50 µL saline alone (n = 14) over 7 minutes. They were euthanized at 24 hours after receiving an MRI scan. All brains were used for immunohistochemistry and immunofluorescence staining.

### 2.3 Subarachnoid hemorrhage model

Endovascular perforation was used to induce SAH as previously described.^15^, ^16^ Rats were anesthetized using 5% isoflurane (VetOne Fluriso; MWI), intubated, and placed on mechanical ventilation. Isoflurane was then titrated between 2.5% and 3%. Core body temperature was maintained at 37.5°C using a heating pad with a feedback‐control mechanism. While in the supine position, the left external carotid artery was transected distally and reflected caudally in reference to the internal carotid artery. A 3‐0‐nylon monofilament suture was inserted into the stump of external carotid artery and advanced distally into the intracranial internal carotid artery until resistance was felt. At that point, the artery at the bifurcation of intracranial internal carotid was perforated to induce SAH. The suture was then removed into the external carotid artery.

### 2.4 Intraventricular injection

Rats received were anesthetized with pentobarbital (50 mg/kg; IP), and the core body temperature kept at 37.5°C using a heating pad. Rats were then positioned in a stereotaxic frame (Kopf Instruments), a 1 mm cranial burr hole was drilled, and a 26‐gauge needle stereotaxically inserted into the right lateral ventricle using the following coordinates: 4.5 mm ventral, 0.6 mm posterior, and 1.6 mm lateral to the bregma. Rats received either saline or thrombin injected using a microinfusion pump (World Precision Instruments). The needle was then removed, bone wax was used to fill the burr hole, and the skin incision sutured closed.

### 2.5 Magnetic resonance imaging (MRI) and ventricle volume measurement

Rats were anesthetized with 2% isoflurane throughout an MRI using a 7.0‐T Varian MR scanner (Varian) with acquisition of T2 fast spin‐echo and T2* gradient‐ echo sequences using 25 coronal slices (0.5 mm thick) with a field of view of 20 × 20 mm and matrix of 256 × 256 mm. Ventricular volume was measured from the frontal horns of the lateral ventricles to the foramen of Luschka using the sum of the ventricular area over all slices and multiplying by section thickness. Image analysis was performed using Image J by a blinded observer. Hydrocephalus was defined as a ventricular volume over + 3 standard deviations (SD) from the mean of the sham animals.

### 2.6 Immunohistochemistry and immunofluorescence staining

Immunohistochemistry was performed as described previously.^17^ In brief, rats were anesthetized using pentobarbital (100 mg/Kg; IP) and then underwent transcardiac perfusion with 4% paraformaldehyde in 0.1 mol/L phosphate‐buffered saline (pH 7.4). Brains were then harvested and kept in 4% paraformaldehyde for 24 hours. Next, they were immersed in 30% sucrose for 3‐4 days at 4°C prior to embedding in a mixture of 30% sucrose and optimal cutting temperature compound (Sakura Finetek Inc; 1:2 ratio) and sectioning on a cryostat (18 µm). Immunohistochemistry was performed using the avidin‐biotin complex technique. The primary antibodies were polyclonal goat anti‐Iba‐1 (ionized calcium‐binding adapter molecule 1, 1:400 dilution; Abcam), monoclonal mouse anti‐MHC II RT 1B (clone number: OX‐6, 1:200 dilution; AbD Serotec), and monoclonal mouse anti‐CD68 (clone number: ED1, 1:100 dilution; Abcam).

Double labeling immunofluorescence was performed on paraffin‐embedded sections. Primary antibodies were added and incubated overnight at 4°C. Sections were then rinsed in PBS prior to incubation at room temperature for 2 hours with the secondary antibodies. The primary antibodies were polyclonal goat anti‐Iba‐1 (ionized calcium‐binding adapter molecule 1, 1:400 dilution; Abcam), monoclonal mouse anti‐MHC II RT 1B (OX‐6, 1:200 dilution; AbD Serotec), and monoclonal mouse anti‐CD68 (ED1, 1:100 dilution; Abcam). Secondary antibodies were Alexa Fluor 594 donkey anti‐mouse IgG (1:500, Invitrogen) and Alexa Fluor 488 donkey anti‐ goat IgG (1:500, Invitrogen). Fluoroshield^™^ with DAPI (F6057) was used for nuclear labeling.

### 2.7 Cell counting and soma size measurement

Immuno‐positive cells were counted on high‐power images (×40 magnification) from a digital camera. Both lateral ventricle choroid plexuses were counted. The number of epiplexus cells was calculated as a percentage of total choroid plexus epithelial cell number. Soma size measurements were determined on high‐power images (×100 magnification). Ten immuno‐positive cells from one sample were randomly chosen to have their soma sizes measured. Cell counts and soma size measurements were performed using Image J. Mean values are reported from measurements that were repeated three times by a blinded observer.

### 2.8 Statistical analysis

Data are expressed as mean ± standard deviation (SD), and comparisons between groups were performed using Student *t*‐test or one‐way ANOVA with a Tukey's post hoc test for multiple comparisons. *P* < .05 was considered to be statistically significant.

## 3 RESULTS

### 3.1 Iba‐1, OX‐6, and CD68 are expressed in choroid plexus epiplexus cells

**Figure 1:**
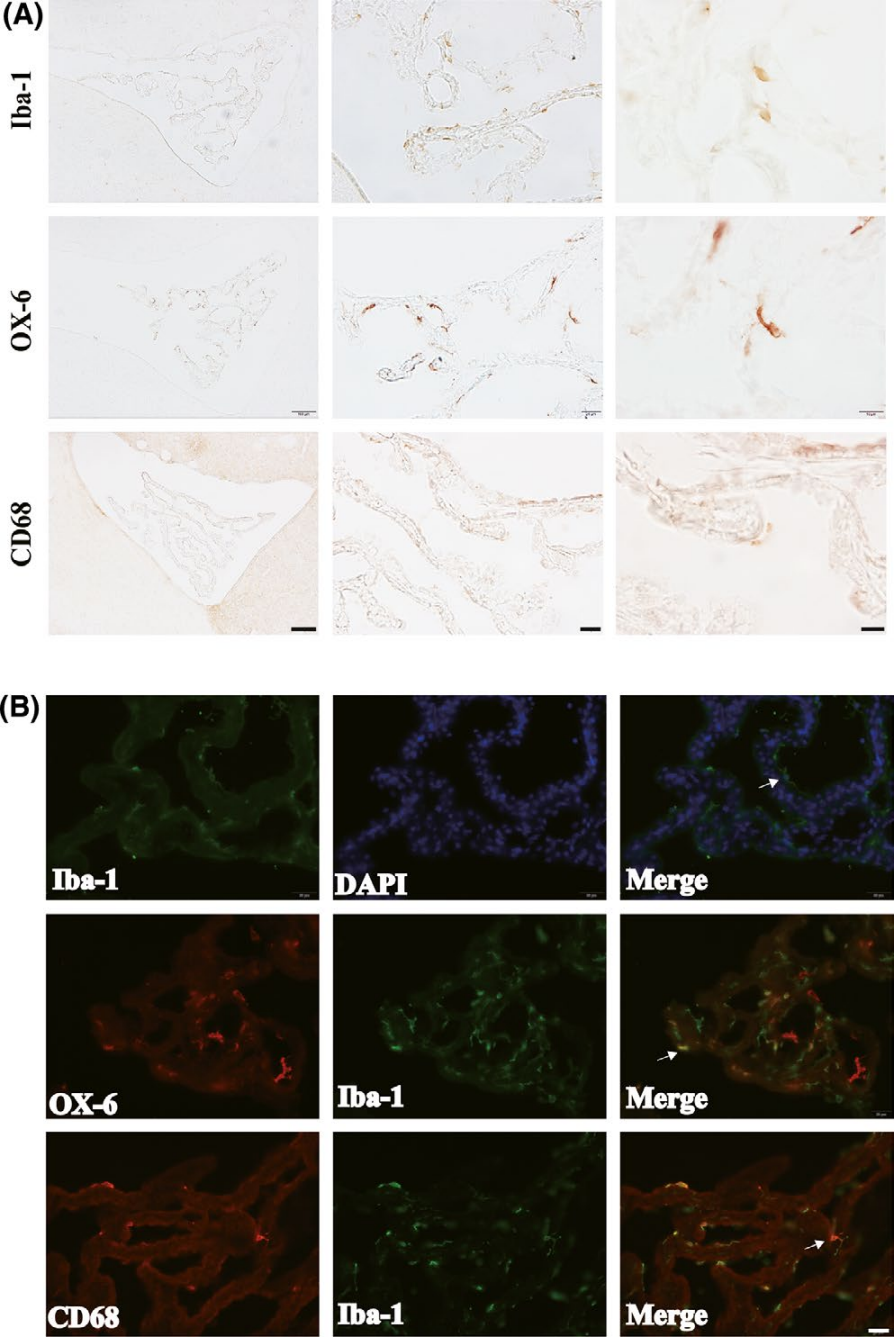
A, Iba‐1, OX‐6, and CD68 immunoreactivity in epiplexus cells of rats at 24 h after sham operation. Scale bar =100 µm in first column, 20 µm in second column, and 10 µm for third column. B, Immunofluorescence staining of Iba‐1 and the double labeling of Iba‐1 with OX‐6 or CD68 in epiplexus cells at 24 h in sham‐operated rats. Scale bar =20 µm

Epiplexus cells are macrophage‐like cells that reside on the apical surface of the choroid plexuses. Iba‐1, OX‐6, and CD68 (three macrophage/microglia markers) immunoreactivity was noted in epiplexus cells at 24 hours in sham rat group (Figure 1A). These cells displayed diverse morphologies ranging from round to stellate. Immunofluorescence double‐staining revealed that almost all CD68‐positive epiplexus cells also stained for Iba‐1. In contrast, most of OX‐6‐positive epiplexus cells did not stain for Iba‐1 (Figure 1B).

### 3.2 Iba‐1‐positive cells increased and enlarged after SAH

**Figure 2:**
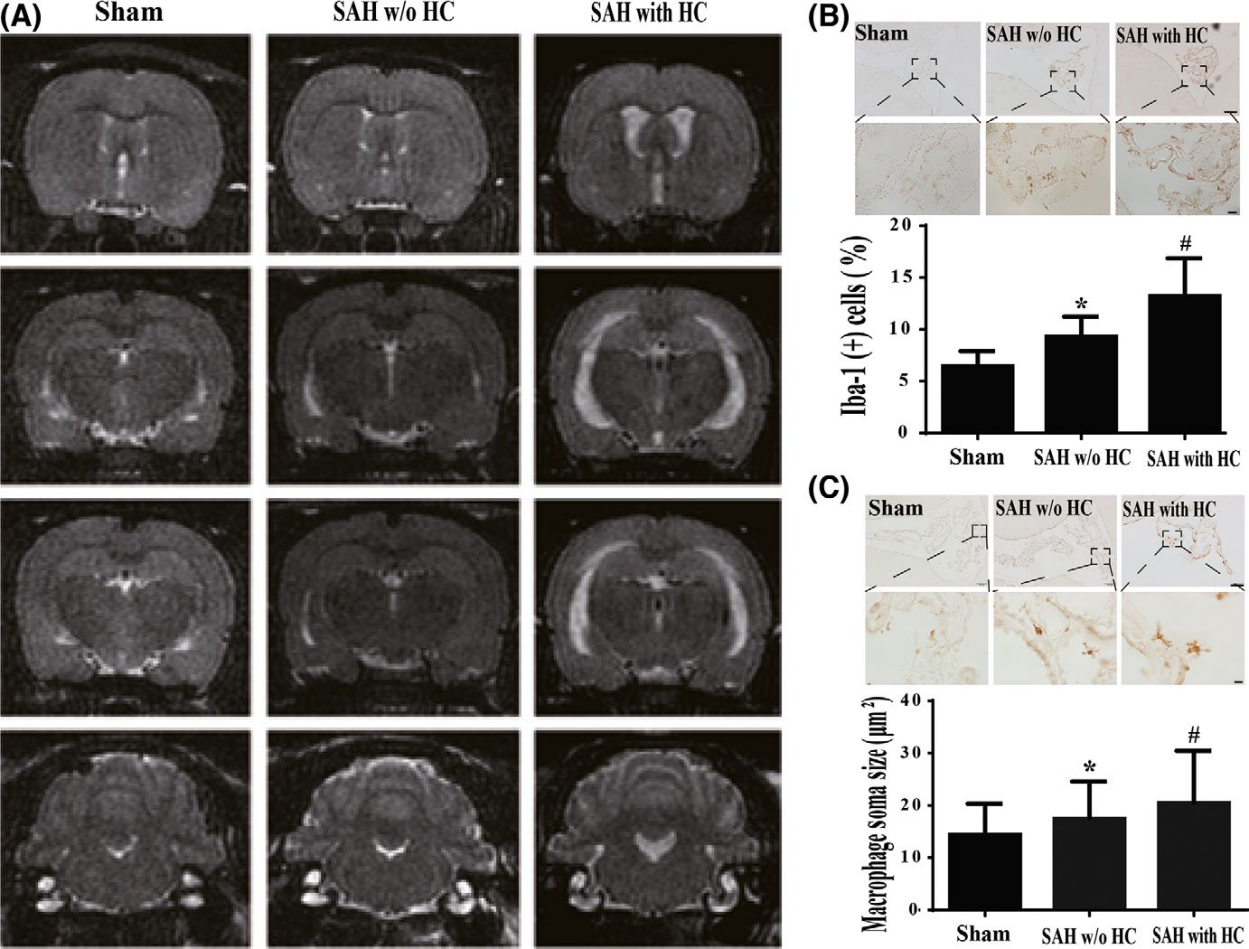
A, Representative examples of T2‐weighted magnetic resonance images of rats in sham, SAH without (w/o) hydrocephalus (HC), and SAH with HC groups at 24 h after procedure. B, Iba‐1 immunoreactivity in the epiplexus cells of rats at 24 h in sham, SAH w/o HC, and SAH with HC groups. Scale bars =100 µm (upper row) and 20 µm (lower row). The number of Iba‐1‐positive cells was quantified relative to the number of choroid plexus epithelial cells. The values are means ± SD; n = 10 in sham group, n = 12 in SAH w/o HC group, and n = 15 in SAH with HC group, *P < .05 vs sham group, #*P* < .01 vs sham group and SAH w/o HC group by one‐way ANOVA. C, Iba‐1‐positive cell soma size in the epiplexus cells of rats at 24 h in sham, SAH w/o HC, and SAH with HC groups. Values are means ± SD; n = 10 in sham group, n = 12 in SAH w/o HC group and n = 15 in SAH with HC group, *P < .05 vs sham group, #*P* < .01 vs sham group and SAH w/o HC group by one‐way ANOVA. Scale bars =100 µm (upper row) and 10 µm (lower row)

Examples of MRIs of rats with and without (w/o) hydrocephalus (HC) after SAH are shown in Figure 2A. In the rats w/o HC, there was an increase in the number of Iba‐1‐positive cells at 24 hours in SAH (9.3 ± 0.6%; n = 10) compared with sham‐operated rats (6.5 ± 0.4%; n = 12, *P* < .05, Figure 2B). This was further magnified in the SAH with HC group (13.2 ± 0.9%; n = 15; *P* < .01 vs w/o HC group; Figure 2B). Additionally, macrophage soma size in the SAH w/o HC group (17.4 ± 0.7 μm^2^; n = 10) was significantly larger in the sham group (14.4 ± 0.5 μm^2^; n = 12, *P* < .05, Figure 2C). Again, this effect was larger in SAH with HC group (20.5 ± 0.8 μm^2^; n = 15, *P* < .01 vs w/o HC group; Figure 2C).

### 3.3 CD68 expression was increased after SAH

**Figure 3:**
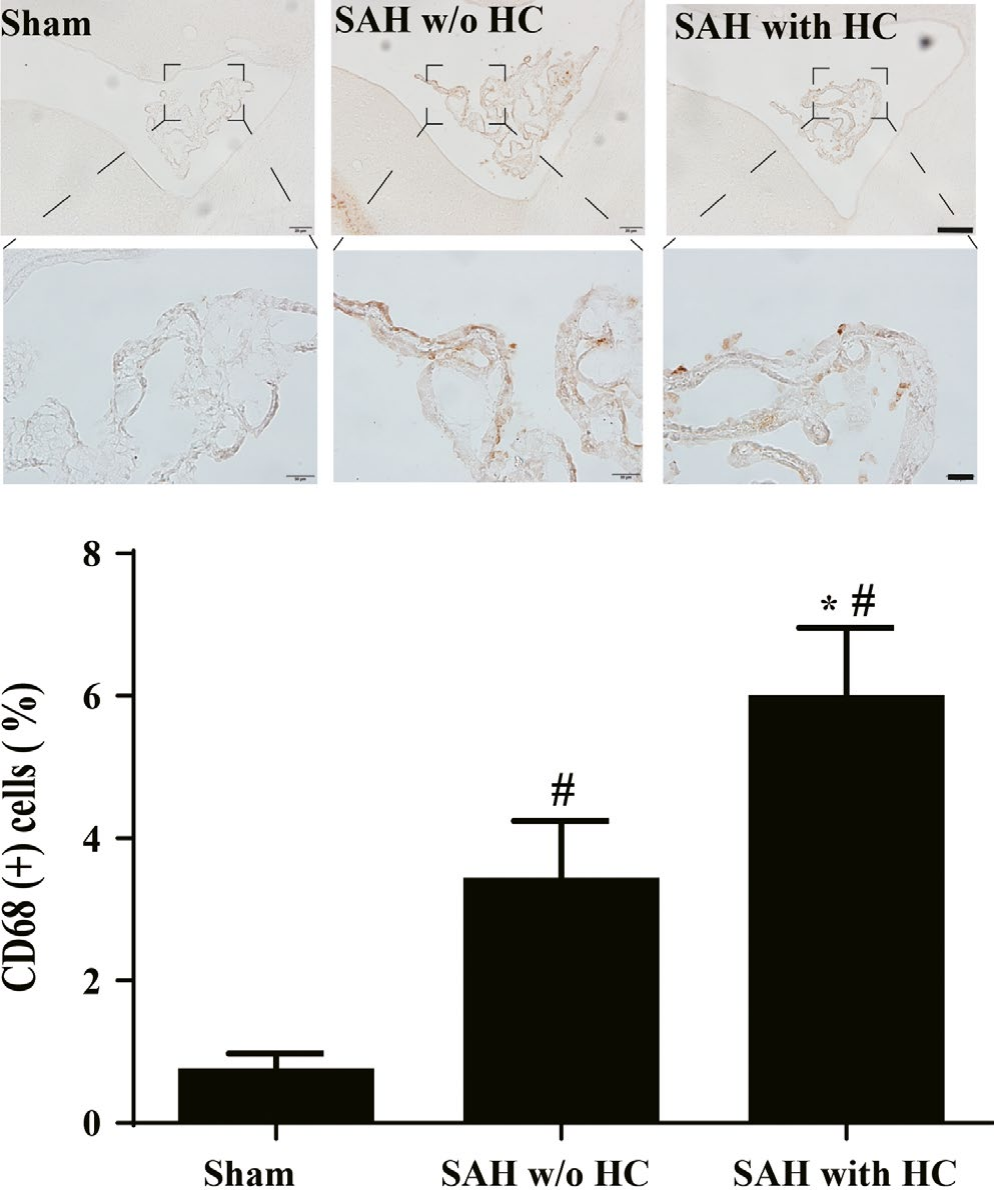
CD68 immunoreactivity in epiplexus cells in sham, SAH without (w/o) hydrocephalus (HC), and SAH with HC groups. The number of CD68‐positive cells was quantified relative to the number of choroid plexus epithelial cells. Values are mean ± SD; n = 7 in sham group, n = 8 in SAH w/o HC group, and n = 9 in SAH with HC groups, #*P* < .01 vs sham group and SAH with HC group, *#*P* < .01 vs sham group by one‐way ANOVA. Scale bar = 100 µm (upper row) and 20 µm (lower row)

The number of CD68‐positive cells was also significantly increased at 24 hours in SAH w/o HC group (3.4 ± 0.3%, n = 8) compared with sham‐operated rats (0.7 ± 0.1%; n = 7, *P* < .01; Figure 3). Again, this effect was magnified in the SAH with HC group (6.0 ± 0.3%, n = 9, *P* < .01 vs w/o HC; Figure 3).

### 3.4 Iba‐1‐positive cells were upregulated and activated after intraventricular thrombin injection

**Figure 4:**
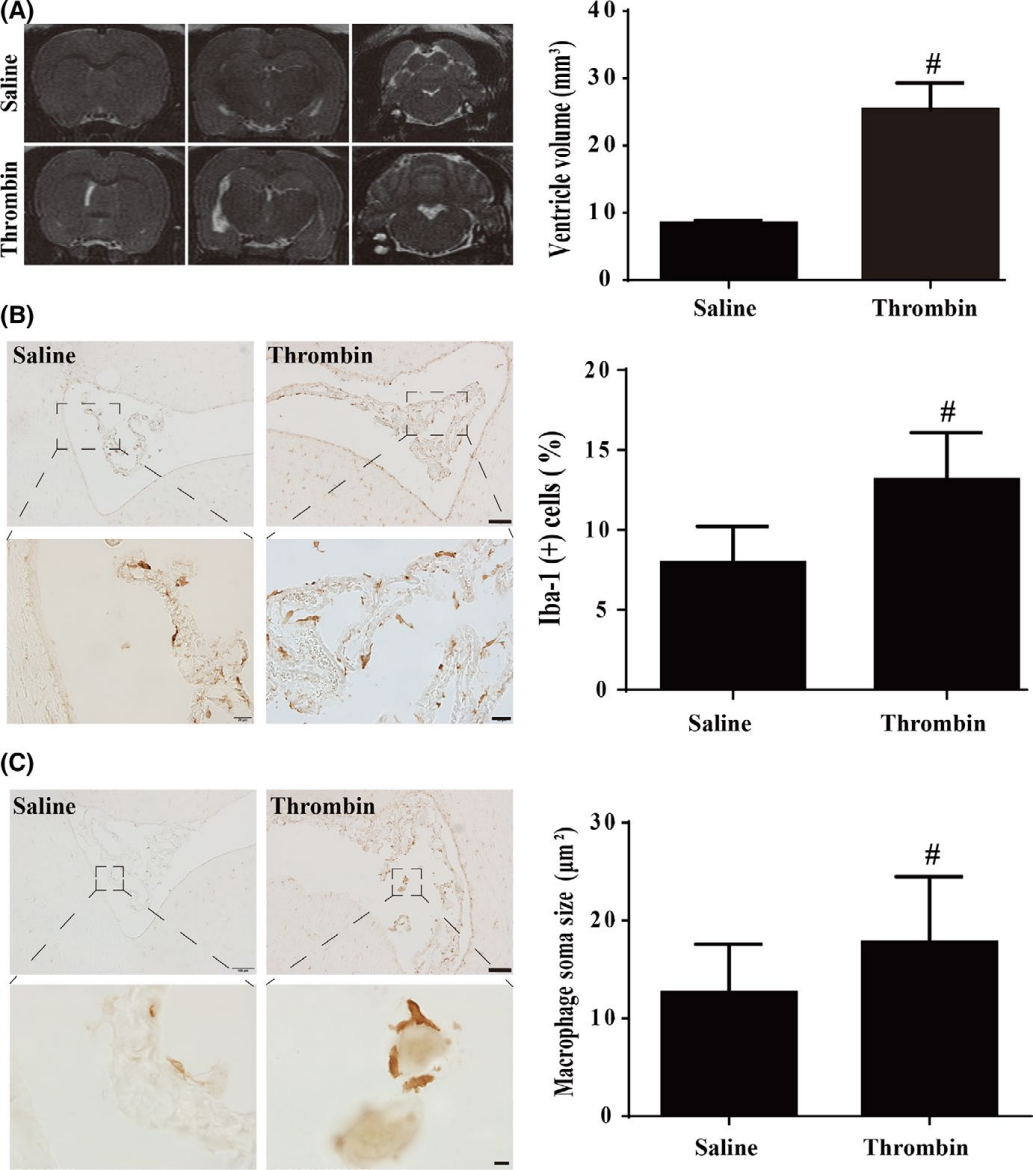
A, Examples of T2‐weighted MRI scan 24 h after intraventricular injection of saline or thrombin. Note the dilated ventricles in the thrombin‐injected rats. Ventricular volume was quantified (bar graph). Values are mean ± SD; n = 11 in saline group and n = 14 in thrombin group, #*P* < .01 vs injected saline group by Student *t*‐test. B, Iba‐1 immunoreactivity in epiplexus cells 24 h after intraventricular injections of saline or thrombin (3U). The number of Iba‐1‐positive cells was calculated and expressed relative to the number of choroid plexus epithelial cells. Values are means ± SD; n = 11 in saline group and n = 14 in thrombin group, #*P* < .01 vs injected saline group by Student *t‐*test. Scale bar = 100 µm (upper row) and 20 µm (lower row). C, Iba‐1‐positive cell soma size at 24 h after injection of 50 µL of saline or thrombin (3U) in saline. Values are means ± SD; n = 11 in saline group and n = 14 in thrombin group, #*P* < .01 vs injected saline group by Student *t*‐test. Scale bar =100 µm (upper row) and 10 µm (lower row)

Intraventricular injection of thrombininduced hydrocephalus in rats as identified on MRI at 24 hours (Figure 4A). It also increased the number of Iba‐1‐positive epiplexus cells (13.2 ± 0.8%, n = 14 vs 7.9 ± 0.7% in saline‐injected group, n = 11, *P* < .01, Figure 4B). In addition, the size of those cells was bigger (17.8 ± 0.5 μm^2^; n = 14) than in saline‐injected rats (12.6 ± 0.4 μm^2^; n = 11, *P* < .01, Figure 4C).

### 3.5 CD68‐positive cells increased after intraventricular thrombin injection

**Figure 5:**
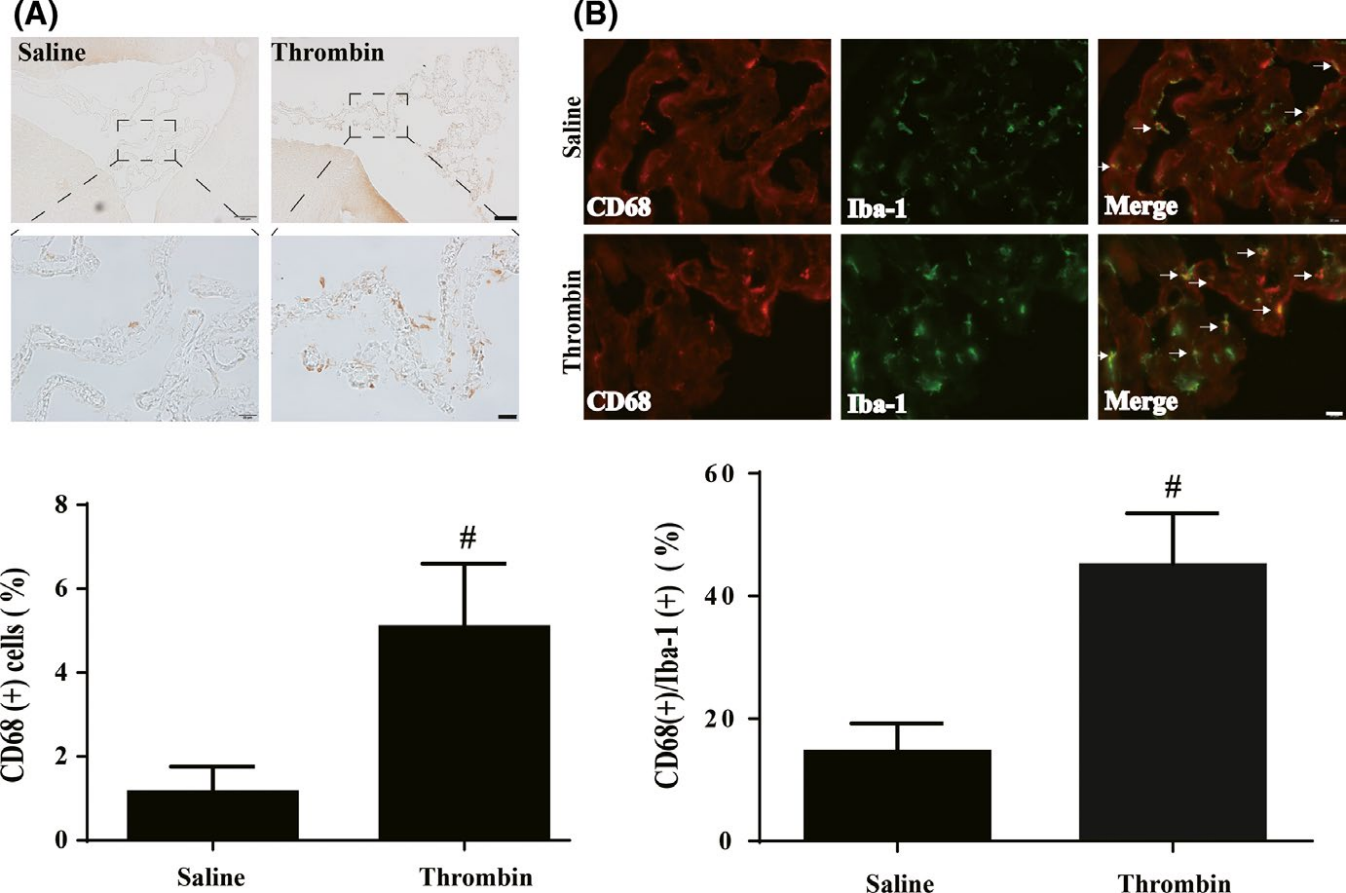
A, CD68 immunoreactivity in epiplexus cells 24 h after intraventricular injection of saline or thrombin (3U). The number of CD68‐positive cells was quantified and expressed relative to the number of choroid plexus epithelial cells. Values are means ± SD; n = 11 in saline group and n = 14 in thrombin group, #*P* < .01 vs saline group by Student *t*‐test. Scale bars =100 µm (upper row), 20 µm (lower row). B, Double‐staining of CD68 and Iba‐1 in epiplexus cells in saline and thrombin groups at 24 h. The percentage of Iba‐1‐positive epiplexus cells that were also CD68 positive also increased with thrombin injection. Values are means ± SD; n = 6, # *P* < .01 vs saline group by Student *t*‐test; scale bar =20 µm

Intraventricular injection of thrombin also increased the number of CD68‐positive epiplexus cells at 24 hours (5.1 ± 0.4%; n = 13 vs 1.1 ± 0.2% in the saline‐injected group, n = 11; *P* < .01, Figure 5A). In addition, the percentage of Iba‐1‐positive cells that were also CD68 positive was increased after thrombin injection (45 ± 3%; n = 6) compared with saline controls (15 ± 2%, n = 6; *P* < .01, Figure 5B).

## 4 DISCUSSION

The main findings of this study are as follows: (a) Iba‐1, CD68, and OX‐6 are expressed in epiplexus cells in sham‐operated rats; (b) induction of SAH increased the numbers of CD68‐ and Iba‐1‐positive cells, as well as Iba‐1‐positive cell soma size at 24 hours in rats; (c) Those effects were greater in rats that developed hydrocephalus after SAH; (d) Intraventricular injection of thrombin in rats also increased the number of Iba‐1‐positive and CD68‐positive cells at 24 hours.

The epiplexus, free‐floating, and supraependymal cells are all considered intraventricular macrophages. Epiplexus cells, also named “Kolmer cells,” were initially detailed in 1921 by Kolmer, residing on the apical surface (CSF‐facing) of the choroid plexus. These cells have been examined by immunohistochemistry and electron microscopy.^8^ There have been several theories regarding the origin of epiplexus cells. However, the most accepted theory is that circulating monocytes become tissue macrophages after reaching connective tissue spaces by migrating from the thin‐walled choroid blood vessels. These macrophages become epiplexus cells after they pass through the choroid plexus epithelium.^18^ These cells are in close proximity to the surface of the choroid plexus epithelium and show different morphologies which range from round to polar and stellate.^19^ Epiplexus cells are mainly associated with antigen presentation, nitric oxide (NO) production, phagocytosis of various foreign bodies, and iron accumulation and are thus considered to have an immunologic role in the brain ventricles.^9^ A previous study found that epiplexus cells express OX‐42, which recognizes complement type 3 receptors (CR3), and OX‐18, which recognizes major histocompatibility complex (MHC) antigens in rats of different ages.^20^ In our study, immunohistochemistry and immunofluorescence staining indicated that Iba‐1, OX‐6, and CD68 were expressed in epiplexus cells of sham‐operated rats. OX‐6 recognizes major histocompatibility complex II (MHC II) antigens as an activated macrophage marker in rat brains, while Iba‐1 is a macrophage marker in many sites in brain, including the choroid plexus in rat.^21^, ^22^ An interesting phenomenon was that most OX‐6‐positive epiplexus cells were different from Iba‐1‐positive epiplexus cells, with only a small portion colocalizing on immunofluorescence staining. The role of these subsets of epiplexus cells requires further investigation.

Subarachnoid hemorrhage resulted in an increase in the number of Iba‐1‐positive and CD68‐positive epiplexus cells on the choroid plexus, as well as an increase in soma size in Iba‐1‐positive cells. This suggests that SAH results in epiplexus cell activation. One potential factor that might participate in such activation is thrombin that is generated during clot formation. Thrombin can activate microglia/macrophages,^13^ and we found that intraventricular injection of thrombin mimicked the effects of SAH, increasing the number of Iba‐1‐positive and CD68‐positive epiplexus cells, and increasing soma size. This suggests that thrombin may play an important role in epiplexus cell activation after SAH.

We previously found that hydrocephalus occurs in about 44% of rats with SAH induced by endovascular perforation^16^ and that thrombin directly contributes to hydrocephalus development after intraventricular hemorrhage.^14^ Several research studies were interested in the macrophages of the cerebrospinal fluid, cerebral cortex, and the basal ganglia after SAH.^23^, ^24^, ^25^ Another study revealed that intraventricular macrophages were activated by inducing hydrocephalus in prenatal rats by injecting pregnant rats with 6‐aminonicotinamide.^26^ Mature microglia/macrophage have highly ramified processes, adopting an “ameboid” morphology with larger, rounder cell bodies during activation.^27^ Interestingly, our data indicate that compared with the SAH without hydrocephalus group, the SAH with hydrocephalus rats have increased epiplexus cell numbers and size. The effect of epiplexus cell activation requires further investigation. These cells become remarkably larger with ruffles and may participate in phagocytosis of erythrocytes in SAH. The cells stained with Iba‐1 and OX‐6 antibodies had enhanced immunoreactivity, suggesting immunoregulatory engagement due to upregulation of phagocytic activity. Their location on the apical surface of the choroid plexus epithelium, also suggests they may modify choroid plexus, the site of the blood‐CSF barrier, function.

CD68 is a classic M1‐like (proinflammatory) phenotype of microglia/macrophage activated in response to cerebral hemorrhage and other neurological conditions.^28^, ^29^ In our study, CD68 expression was very low in both the sham and intraventricular injection of saline groups. However, it significantly increased after SAH and intraventricular injection of thrombin in rats. Iba‐1 and CD68 are two widely used markers to identify microglia/macrophage in human and animal brains.^30^, ^31^ In the present study, the percentage of Iba‐1‐positive epiplexus cells that were CD68 positive was significantly increased by thrombin. This phenomenon suggests that M1 polarization becomes a more predominant response, thus causing more inflammatory changes, eventually leading to hydrocephalus development.

In summary, this study reveals that SAH induces choroid plexus epiplexus cell activation, an effect mimicked by intraventricular thrombin. There was a positive correlation between such activation and hydrocephalus development. Whether epiplexus cell activation participates in, or is a result of, the hydrocephalus development needs further investigation.

## CONFLICT OF INTEREST

We declare that we have no conflict of interest.
